# *MYOD1* inhibits avian adipocyte differentiation via miRNA-206/*KLF4* axis

**DOI:** 10.1186/s40104-021-00579-x

**Published:** 2021-05-05

**Authors:** Zheng Wang, Qiangsen Zhao, Xiaoqin Li, Zhongtao Yin, Sirui Chen, Sen Wu, Ning Yang, Zhuocheng Hou

**Affiliations:** 1 National Engineering Laboratory for Animal Breeding and Key Laboratory of Animal Genetics, Breeding and Reproduction, MARA, College of Animal Science and Technology, China Agricultural University, Yuanmingyuan West Road No. 2, Beijing, 100193 China; 2 State Key Laboratory of Agrobiotechnology, College of Biological Sciences, China Agricultural University, Yuanmingyuan West Road No. 2, Beijing, 100193 China

**Keywords:** Adipocyte differentiation, Avian, CRISPR/Cas9, miR-206/*KLF4* axis, *MYOD1*

## Abstract

**Background:**

A considerable number of muscle development-related genes were differentially expressed in the early stage of avian adipocyte differentiation. However, the functions of them in adipocyte differentiation remain largely known. In this study, the myoblast determination protein 1 (*MYOD1*) was selected as a representative of muscle development. We investigated its expression, function, and regulation in avian adipocyte differentiation.

**Results:**

The expression of *MYOD1* decreased significantly in the early stage of avian adipocyte differentiation. CRISPR/Cas9-mediated deletion of *MYOD1* induced adipocyte differentiation, whereas over-expression of *MYOD1* inhibited adipogenesis. The mRNA-seq data showed that *MYOD1* could perturb the lipid biosynthetic process during differentiation. Our results showed that *MYOD1* directly up-regulates the miR-206 expression by binding the upstream 1200 bp region of miR-206. Then, over-expression of miR-206 can inhibit the adipogenesis. Furthermore, *MYOD1* affected the expression of endogenous miR-206 and its target gene Kruppel-like factor 4 (*KLF4*), which is an important activator of adipogenesis. Accordingly, the inhibition of miR-206 or over-expression of *KLF4* could counteract the inhibitory effect of *MYOD1* on adipocyte differentiation.

**Conclusions:**

Our results establish that *MYOD1* inhibits adipocyte differentiation by up-regulating miR-206 to suppress the *KLF4* expression. These findings identify a novel function of *MYOD1* in adipocyte differentiation, suggesting a potential role in body-fat distribution regulation.

**Supplementary Information:**

The online version contains supplementary material available at 10.1186/s40104-021-00579-x.

## Background

Adipocytes are unique in the quantity of lipids that they can store, the rapid release of these calories, and protein for use by other organs, which can profoundly affect our health [1]. Avian has been utilized as a good animal model for studying basic adipogenesis mechanisms, which can be directly used for the genetic improvement of avian fat deposition [2]. Adipogenesis is driven by an increase in adipocyte cell size (hypertrophy) or number (hyperplasia) [3]. Adipocyte differentiation is regulated by an elaborate network of transcription factors, and understanding the underlying transcriptional networks is relevant and timely both from a basic and medical research perspective [4].

Several studies and observations showed that fat deposits in the muscles will cause the loss of muscle quality and are more likely to induce metabolic diseases in various animals [5–8]. Adipose and muscle tissues originate from mesenchymal stem cells (MSCs) [9–12], which are the multipotent and relevant targets for therapies aiming to enhance tissue regeneration [13]. In response to lineage-specific inducers, MSCs from different depots can differentiate into many different, mutually exclusive lineages, including the adipocyte and myoblast lineages [14–16]. It has been shown that peroxisome proliferator-activated receptor γ (*PPARγ*) and myoblast determination protein 1 (*MYOD1*) are the master regulators of adipogenesis and myogenesis, respectively [17, 18]. At the same time, the *MYOD1*-driven and *PPARγ*-driven differentiation programs are mutually exclusive [19–21]. However, the functional contributions of muscle development genes to adipocyte differentiation remain largely unexplored.

The microRNA-206 (miR-206) is a vertebrate-specific miRNA, which belongs to the skeletal muscle-specific myomiR family (myomiRs) along with miR-1, miR-133a, miR-133b. It has been confirmed to be involved in the pathogenesis of many diseases, including heart failure, chronic obstructive pulmonary disease, and various types of cancers [22, 23]. In chicken, miR-206 is significantly associated with broiler birthweight [24]. Various TFs essential for skeletal muscle development have been shown to regulate miR-206 expression during myogenic differentiation, such as *MYOD1*, myogenic factor 5 (*MYF5*), myogenin (*MYOG*), and myocyte enhancer factor 2C (*MEF2C*) [25–27]. In mammals, miR-206 promotes apoptosis, induces cell cycle arrest, and inhibits cell migration and adipocyte differentiation by targeting *c-MET* and its downstream PI3K/AKT pathway [28, 29]. However, the role of miR-206 in avian adipocyte differentiation still remains unclear.

Kruppel-like factor 4 (*KLF4*) is an evolutionarily conserved zinc finger-containing transcription factor which is also known for being one of four key factors required for inducing pluripotent stem cells. *KLF4* has been shown to be induced very early following the induction of adipogenesis, and acted as an activator of adipogenesis by inducing CCAAT enhancer binding proteins β (*C/EBPβ*) expression [30], which then trans-activate *C/EBPα* and *PPARγ* [31, 32]. Studies have shown that knocking down *KLF4* suppresses adipocyte differentiation [30]. More recent studies have shown that the ability of A2b adenosine receptor (*ADORA2B*) to block adipocyte development has been shown to be dependent on its ability to modulate *KLF4* expression [33].

Our previous work found many muscle development genes involved in the early regulation of adipocyte differentiation [34]. In this study, *MYOD1*, a representative of muscle development, was significantly down-regulated in the early stage of avian adipocyte differentiation. Studies of loss-of-function and gain-of-function demonstrated that *MYOD1* is a key repressor of adipocyte differentiation by interacting with miR-206/Kruppel-like factor 4 (*KLF4*) axis. The mRNA-seq showed that over-expression of *MYOD1* in adipocytes inhibits the expression of most lipid biosynthesis genes and also promotes the expression of some myogenic genes. Our findings imply that a novel function of *MYOD1* in adipocyte differentiation. Thus, affecting *MYOD1* might represent a viable strategy to improve fat ratio to muscle in avian.

## Materials and methods

### Plasmid construction

*MYOD1*- knock-out and knock-in plasmids: Using the *MYOD1* sequence obtained from the NCBI database (Accession: NC_006092.5), we designed gRNA sequences targeting exon1 of *MYOD1*, known as sgRNA1: CGACCCGTGCTTCAACACGT and sgRNA2: GCGGCTCAGCAAGGTCAACG. We synthesized the oligo-DNAs corresponding to these gRNAs. We annealed them to a T7 promoter-driven *Cas9* and to a U6 promoter-driven gRNA vector in order to obtain two gRNA-expressing plasmids. In order to construct the *MYOD1*-over-expression vector, the full-length coding sequence of *MYOD1* (NCBI Reference Sequence: NM_204214.2) was amplified from chicken subcutaneous adipose cDNA by PCR, and cloned into the CMV promoter-driven piggyBac and an EF1α promoter-driven *GFP* plasmid by replacing *GFP* using *Eco*RI and *Sal*I (New England Biolabs, Ipswich, MA, USA).

pmirGLO dual-luciferase reporters: The 3′-UTR fragment of *KLF4* (NCBI Reference Sequence: XM_004949369.3) containing the binding sites were amplified by PCR from chicken subcutaneous adipose cDNA and then cloned into pmirGLO vector. The mutant vectors were constructed by PCR mutagenesis. Six seed sequences were successfully mutated from CATTCC to GTGAAG for the *KLF4*-3′-UTR vector.

Gene over-expression vector: The *MYOD1* and *KLF4* over-expression vector was constructed according to the user manual of the Easy Ligation Kit (Sidansai, Shanghai, China). *MYOD1* and *KLF4* coding sequence (NCBI Reference Sequence: NM_204214.2 and XM_004949369.3) were amplified from chicken subcutaneous adipose cDNA by PCR. The PCR product was cloned into the pcDNA3.1 vector. The successful *MYOD1* and *KLF4*, over-expression vector, was confirmed by DNA sequencing.

miR-206 promoter-reporter plasmid: A 1876 bp fragment of the miR-206 promoter was isolated by PCR using the primers listed in Table S1. The PCR product was digested with *Kpn*I and *Sma*I restriction enzymes (Takara, Otsu, Japan), then the insertion was ligated into the pGL4.10 vector (Promega, Madison, WI, USA) to create the expression vector pGL4.10_−1876 bp. The pGL4.10_−1876 bp was subjected to be sequenced, and this construct was used as a template, and pGL4.10_-1234 bp was isolated by PCR. All cloning plasmids were confirmed by sequencing.

### RNA extraction, cDNA synthesis, and quantitative real-time PCR

According to the manufacturer’s instruction, the total RNA was isolated from the cells using RNAiso reagent (Takara, Otsu, Japan). According to the manufacturer’s manual, the reverse transcription reaction for mRNA was performed with PrimeScript RT reagent Kit (Perfect Real-Time) (Takara, Otsu, Japan). The reverse transcription reaction for miRNA was using miRNA First-Strand cDNA Synthesis SuperMix (Transgen, Beijing, China). The specific qRT-PCR Primer of mRNA and miRNA were designed using Primer 3 software (version 0.4.0, Howard Hughes Medical Institute). Primer sets are listed in Table S1. The qPCR reaction, which contained KAPA SYBR FAST qPCR Kit (KAPA Biosystems, MA, USA), was carried out in ABI-7500 PCR machine (Applied Biosystems, MA, USA) following a standard thermal protocol as previously described [35]. All reactions were run in triplicate.

### Cell culture

A cell line of immortalized chicken preadipocytes (ICPs) [36] was cultured in DMEM/F12 (Gibco, Gaithersburg, MD, USA) supplemented with 10% fetal bovine serum (Hyclone, Logan, UT, USA), and 0.2% penicillin/streptomycin (Invitrogen, Carlsbad, CA, USA). To induce ICPs differentiation, we added 160 μmol/L sodium oleate (Sigma Life Science, St. Louis, MO, USA) to the medium [37].

For *MYOD1*^OE^ and *MYOD1*^KO^ cell selection, ICPs were seeded in 6-well plates for further transfection using Lipofectamine 3000 (Invitrogen, Carlsbad, CA, USA). After a 48-h recovery period, the cells were supplemented with 3 μg/mL of puromycin (Sigma-Aldrich, MO, USA) in the culture medium for 12 days until clone formation. Cells were harvested using 0.25% trypsin/EDTA (Gibco, Gaithersburg, MD, USA), and the cell density was calculated using a handheld automated cell counter (Millipore, Darmstadt, Germany). Single cells were plated in each well of a 96-well plate by limiting dilution and then cultured for 10 d in the cell culture medium. The medium was replaced every 4 d. Confluent cell colonies were propagated and genotyped by PCR and sequencing.

### Transfections

Transfections were performed with Lipofectamine 3000 reagent (Invitrogen, Carlsbad, CA, USA) according to the manufacturer’s direction. Nucleic acids were diluted in OPTI-MEM Medium (Gibco, Gaithersburg, MD, USA). All experiments were carried out at least three times independently.

### Oil red O staining and quantification

The cells were washed with PBS and fixed in 4% formaldehyde for 10 min. The cells were then stained with Oil-Red-O working solution (Solarbio, Beijing, China) according to the manufacturer’s manual. After another wash with PBS, the cell nuclei were counterstained with Hoechst 33342 (Solarbio, Beijing, China). Images were acquired by inverted fluorescent microscope (Nikon) with 20× objective lens (200× magnification), and quantified by Image J software (National Institutes of Health, Bethesda, MD, USA). Particle number was quantified with the analysis particles functions in threshold single sections with size (pixel^2^) setting from 0.1 to 10, and circularity from 0.1 to 1. The Oil-Red-O dyes were then extracted in isopropanol solution containing 4% Nonidet P-40 and quantified by NanoDrop 2000C spectrophotometers (Thermo Fisher Scientific, San Jose, CA, USA) at 510 nm.

### RNA oligonucleotides

The miR-206 mimics, negative control (NC) mimic, miR-206 inhibitors, and NC inhibitor were all purchased from GenePharma (GenePharma, Shanghai, China).

### Dual-luciferase reporter assay

For the promoter activity assays, ICPs were cotransfected with reporter plasmid and *MYOD1* over-expression vector or control vector. The TK-Renilla reporter was also cotransfected to each sample as an internal control using the Lipofectamine 3000 reagent (Invitrogen, Carlsbad, CA, USA) in 48-well plates. The miRNA target verification assay was also performed in ICPs. Wild-type or mutant *KLF4*-3′-UTR dual-luciferase reporter (200 ng) and miR-206 mimic or NC mimic (50 nmol/L) were cotransfected into ICPs. After 48 h transfection, cells were washed by PBS twice, and the activities of Firefly and Renilla luciferase were measured according to the manual of Luc-pair Duo-Luciferase Assay Kit 2.0 (GeneCopoeia, Rockville, MD, USA). All the data were acquired by averaging the results from three independent repeats.

### Western blot

Cultured cells were washed with PBS and homogenized with RIPA buffer (Beyotime, Jiangsu, China) containing protease inhibitor cocktail (Beyotime, Jiangsu, China). Protein concentrations were determined using the BCA Protein Assay Kit (Beyotime, Jiangsu, China). Proteins were denatured and subjected to 10% polyacrylamide gel and transferred to methanol-activated PVDF membranes. Blots were probed using the primary antibodies: mouse anti-*MYOD1* (1:500; Santa Cruz Biotechnology, USA, Cat# sc-377460), rabbit anti-*KLF4* (1:500; Bioss, Beijing, China, Cat# 52850R), mouse anti-*GAPDH*, (1:5000; Bioworld, St Louis Park, MN, USA, Cat#MB001), overnight at 4 °C. After 1 h incubation with anti-mouse or anti-rabbit HRP-conjugated second antibody (1:5000, Bioss, Beijing, China, Cat# 40296G, 40295G). Immunodetection was performed using enhanced chemiluminescence (ECL) Western blotting substrate (Beyotime, Jiangsu, China), and was detected with FluoChem R imaging system (ProteinSimple, CA, USA).

### RNA-seq analysis

Raw reads were trimmed to remove adapters and low-quality reads, with Trimmomatic (version 0.39) [38]. Trimmed reads were mapped to the chickenreference genome (Ensembl release 100:ftp://ftp.ensembl.org/pub/release100/fasta/gallus_gallus/dna/Gallus_gallus.GRCg6a.dna.toplevel.fa.gz) using HISAT2 [39]. Read counts for each gene were calculated using Stringtie (v.2.1.2) and normalized by library sequencing depth using the R package DESeq2 (v.1.28.1) after filtering the gene with low expression [40]. We used the DEGSeq2 (v.1.28.1) package to identify DEGs between *MYOD1*^NC^, *MYOD1*^KO^, and *MYOD1*^OE^ cells at different days (day 0 and day 5). Therefore, samples were excluded from further analysis due to their low global Pearson correlation with the other repeat samples (*R*^2^ < 0.95). Genes with |log_2_FC| ≥ 0.585 (or ≥ 1) and the Benjamini & Hochberg (BH) adjusted *P*-value (adjusted-*P* value) < 0.05 were considered as differentially expressed genes.

### Functional enrichment and prediction of miRNA target genes

Genes were annotated with gene Symbols from the Uniprot database for functional annotation. Gene Ontology (GO) analysis of the enriched genes was performed using the web-based Metascape [41] (a gene annotation & analysis resource, http://metascape.org/gp/index.html#/main). Putative miRNA targets for miR-206 were predicted by online software, TargetScan (version 7.2, http://www.targetscan.org/), miRDB (http://mirdb.org/) as well as miRTarBase (http://mirtarbase.cuhk.edu.cn/) to choose target genes for validation.

### Statistical analysis

Each experiment was repeated three times, and all results are represented as the mean ± SD. Three independent sample t-test was used to perform the statistically significant difference between groups. The level of significance was presented as ∗ (*P* < 0.05).

## Results

### *MYOD1* is a repressor of adipocyte differentiation

**Fig. 1:**
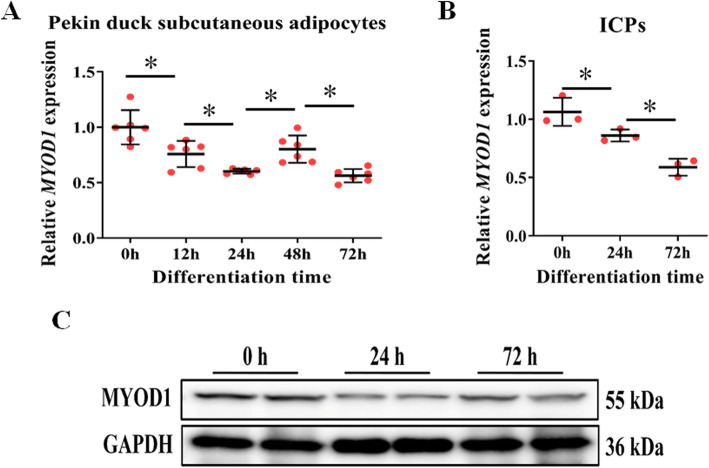
*MYOD1* is down-regulated in the early stage of avian adipocyte differentiation. **a, b** *MYOD1* mRNA levels in different time points during the differentiation of Pekin duck subcutaneous preadipocytes (mRNA-seq) and ICPs (qPCR). **c** *MYOD1* protein levels at different time points during ICPs differentiation were determined by Western blotting. Data are shown as mean ± SD of three biological replicates. The *t*-test was used to analyze the statistical differences between groups. *, *P* < 0.05

In our previous study [34], we found that the expression of *MYOD1* is significantly down-regulated after the beginning of Pekin duck adipocyte differentiation (Fig. 1a). To further understand the relationship between *MYOD1* and adipocyte differentiation, we detected its expression in adipogenic differentiation of ICPs by qPCR (Fig. 1b) and Western blot (Fig. 1c). *MYOD1* was also significantly down-regulated its expression after adipocyte differentiation in chicken. These results indicate that *MYOD1* is involved in the avian adipocyte differentiation process.

**Fig. 2:**
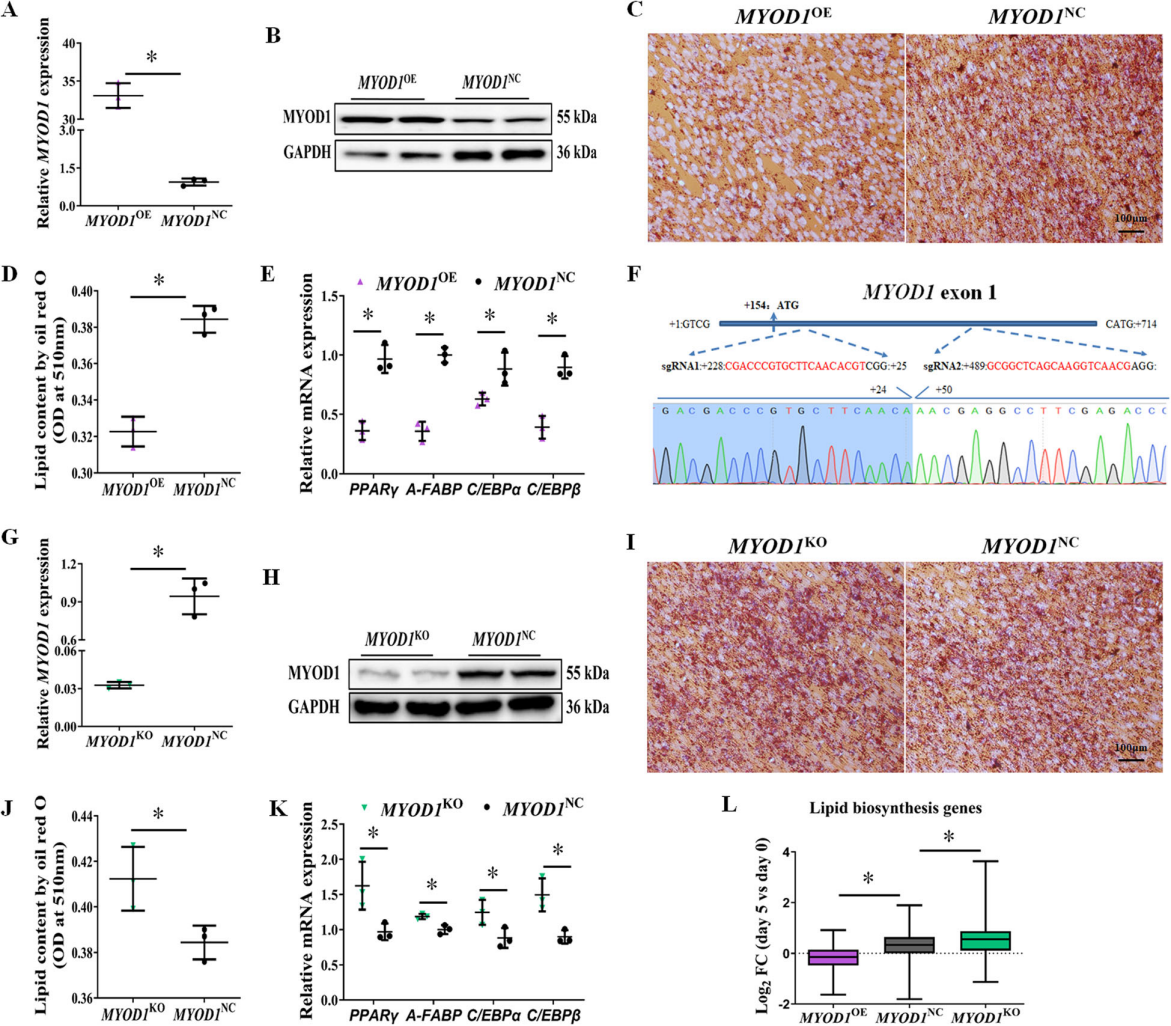
*MYOD1* is a repressor of avian adipocyte differentiation. **a, b** *MYOD1*^OE^ cell significantly promoted *MYOD1* mRNA and protein expression in ICPs. **c** Representative images of *MYOD1*^OE^ cells reduced the lipid droplet formation by oil red O staining on day 3. **d** Comparison of the lipid droplet content of *MYOD1*^OE^ and *MYOD1*^NC^ cells obtained by oil red O staining and extraction methods. **e** mRNA levels of adipocyte genes *PPARγ*, *A-FABP*, *C/EBPα*, and *C/EBPβ* were analyzed with qPCR. **f** Upper part: schematic diagram of *MYOD1* exon1 region and the two targeting loci of *MYOD1* sgRNA (red). Lower part: DNA sequence map around the targeting locus of the cleaved band amplified from ICPs transfected with both sgRNAs. **g, h** *MYOD1*^KO^ significantly reduced *MYOD1* mRNA and protein expression in ICPs. **i** Representative images of *MYOD1*^KO^ cells promoted the lipid droplet formation by oil red O staining on day 3. **j** Comparison of the lipid droplet content of *MYOD1*^OE^ and *MYOD1*^NC^ cells obtained by oil red O staining and extraction methods. **k** mRNA levels of adipocyte marker genes were analyzed with qPCR. **l** Compare the fold change of known lipid biosynthesis genes in *MYOD1*^OE^, *MYOD1*^NC^, and *MYOD1*^KO^ cells on day 5 compared to day 0. Data are shown as mean ± SD of three biological replicates. The *t*-test was used to analyze the statistical differences between groups. *, *P* < 0.05

To further determine the roles of *MYOD1* in avian adipocyte differentiation, we first performed gain-of-function experiments by using piggyBac delivery [42] of *MYOD1* into ICPs. As shown in Fig. 2a and Fig. 2b, transduced *MYOD1* clone can significantly induce *MYOD1* expression relative to cells transduced with a control vector. Strikingly, over-expression of *MYOD1* in ICPs (*MYOD1*^OE^) significantly blocked adipogenesis (Fig. 2c, d), as shown by oil red O staining of neutral lipids (Fig. S1a, b). Adipocyte markers, such as *PPARγ*, *A-FABP*, *C/EBPα,* and *C/EBPβ* (Fig. 2e), were significantly decreased in *MYOD1*^OE^ cells.

We further sought confirmation of *MYOD1* anti-adipogenic activity through loss-of-function studies, in which we would predict enhanced adipose conversion. We transfected ICPs with two single guide RNAs (sgRNAs) targeting the exon 1 of *MYOD1* and a Cas9 vector. We established *MYOD1*-knock-out ICP Clones (*MYOD1*^KO^) with 260-bp frame-shifting deletions in *MYOD1* (Fig. 2f), indicating that both gRNAs work efficiently with Cas9 to edit *MYOD1*. The mutation of *MYOD1* also dramatically down-regulated its expression relative to cells transfected with an only Cas9 vector (Fig. 2g, h). *MYOD1*^KO^ cells demonstrated enhanced adipogenic potential, including greater lipid accumulation (Fig. 2i, j; Fig. S2a, b), and increased the expression of adipocyte marker genes (Fig. 2k).

### *MYOD1* perturb the lipid biosynthetic process

To further understand the effect of *MYOD1* on adipogenesis, we performed mRNA-Seq experiments in *MYOD1*^OE^, *MYOD1*^NC^, *MYOD1*^KO^ cells prior to differentiation (day 0) and day 5 after differentiation. In total, 886 and 1335 differentially expressed genes (DEGs) were up-regulated and down-regulated in *MYOD1*^OE^ cells compared to *MYOD1*^NC^ cells on day 0, respectively (Table S2, fold change > 2; adjusted-*P* value < 0.05). GO analyses for DEGs up-regulated in *MYOD1*^OE^ cells are enriched for development processes such as blood vessel development, muscle structure development, and positive regulation of muscle tissue development. In contrast, DEGs down-regulated in *MYOD1*^OE^ cells are enriched for extracellular matrix organization, metabolism process, and cell morphogenesis involved in differentiation (Table 1, Table S3). Also, 256 and 971 DEGs were up-regulated and down-regulated in *MYOD1*^KO^ cells compared to *MYOD1*^NC^ cells on day 0, respectively (Table S2, fold change > 2; adjusted-*P* value < 0.05). As expected, GO analysis for genes down-regulated in *MYOD1*^KO^ cells also are enriched for striated muscle cell differentiation and muscle system process. In contrast, genes up-regulated in *MYOD1*^KO^ cells are enriched in the adipogenesis related pathway, including cell morphogenesis in differentiation, regulation of MAPK cascade, and positive regulation of lipid metabolic process (Table 1, Table S3). These results indicate that although differentiation has not yet begun, the knock-in or knockout of *MYOD1* alone seems to have affected preadipocyte characteristics.

**Table 1:** GO enrichment analysis of DEGs in *MYOD1*^OE^, *MYOD1*^NC^, and *MYOD1*^KO^ cells

	Up-regulated	Gene count	Log (q-value)	Down-regulated	Gene count	Log (q-value)
*MYOD1*^OE^ vs. *MYOD1*^KO^ (day 0)	blood vessel development	74	−11.19	cell involved in differentiation	25	−1.35
	muscle structure development	64	9.51	extracellular matrix organization	38	−1.35
	heart development	56	−8.84	ATP metabolic process	21	−1.35
	actin cytoskeleton organization	60	−7.72	inorganic cation transmembrane transport	36	−1.12
	muscle tissue development	41	−6.50	developmental growth	30	−0.66
*MYOD1*^KO^ vs. *MYOD1*^NC^ (day 0)	regulation of cell morphogenesis	18	−3.09	regulation of system process	44	−8.02
	positive regulation of cell development	17	−2.34	skeletal system development	35	−5.05
	cell involved in differentiation	19	−2.19	muscle structure development	37	−3.82
	regulation of MAPK cascade	18	−1.89	heart development	34	−3.82
	regulation of lipid metabolic process	11	−1.09	muscle system process	29	−3.66
*MYOD1*^OE^ day 5 vs. day 0	nuclear division	70	−13.19	actin cytoskeleton organization	146	−23.46
	ncRNA metabolic process	78	−10.61	heart development	128	−21.58
	DNA replication	48	−9.70	skeletal system development	118	−20.87
	regulation of cell cycle process	83	−5.79	extracellular structure organization	95	−16.49
	ribosomal large subunit biogenesis	17	−4.12	muscle structure development	126	−15.92
*MYOD1*^NC^ day 5 vs. day 0	nuclear division	59	−21.11	extracellular matrix organization	46	−11.38
	cell division	71	−21.11	heart development	52	−8.01
	lipid biosynthetic process	40	−2.74	muscle structure development	55	−7.35
	regulation of MAPK cascade	36	−1.33	skeletal system development	45	−6.29
	glycerophospholipid metabolic process	19	−1.72	regulation of lipid metabolic process	35	−4.68
*MYOD1*^KO^ day 5 vs. day 0	cell division	68	−6.78	extracellular structure organization	66	−8.36
	protein autophosphorylation	22	−4.05	DNA replication	44	−4.73
	mitotic nuclear division	46	−4.02	skeletal system development	61	−3.78
	lipid biosynthetic process	40	−2.74	phospholipid metabolic process	53	−3.78
	regulation of cell cycle process	64	−2.13	muscle structure development	56	−0.89

To better understand the effect of *MYOD1* on adipogenesis, we re-analyzed the DEGs before and after adipogenic differentiation in *MYOD1*^KO^, *MYOD1*^NC,^ and *MYOD1*^OE^ cells. A total of 2457, 781, and 1636 DEGs were significantly up-regulated on day 5 of differentiation of *MYOD1*^OE^, *MYOD1*^NC,^ and *MYOD1*^KO^ cells compared to day 0, respectively (Table S4, fold change > 1.5; adjusted-*P* value < 0.05). The up-regulated DEGs of the three adipocyte lines on day 5 were significantly enriched in cell division and DNA replication, which is an essential step in adipocyte differentiation. At the same time, down-regulated DEGs are also significantly enriched in muscle structure development (Table 1, Table S5), indicating that muscle development-specific genes are also considerably suppressed during chicken adipocyte differentiation. Notably, we found that the lipid biosynthetic process was only enriched in the DEGs up-regulated on day 5 in *MYOD1*^KO^ and *MYOD1*^NC^ cells, but not in *MYOD1*^OE^ cells (Table 1, Table S5). Furthermore, to test whether *MYOD1* over-expression impacts the lipid biosynthetic process in a statistical threshold-independent manner, we directly compared expression fold-changes of known lipid biosynthesis genes (from Genecards database: Pathcards: Fatty Acyl-CoA Biosynthesis & Triglyceride Biosynthesis) during adipocyte differentiation and found that most lipid biosynthesis genes tended to be up-regulated on day 5 of *MYOD1*^KO^ cells, but down-regulated in *MYOD1*^OE^ cells (Fig. 2l, Table S6), which consistent with observations from our phenotype-profiling experiments and indicating that *MYOD1* is a repressor for the adipogenesis.

### The expression of miR-206 can be significantly induced in *MYOD1*^OE^ cells

**Fig. 3:**
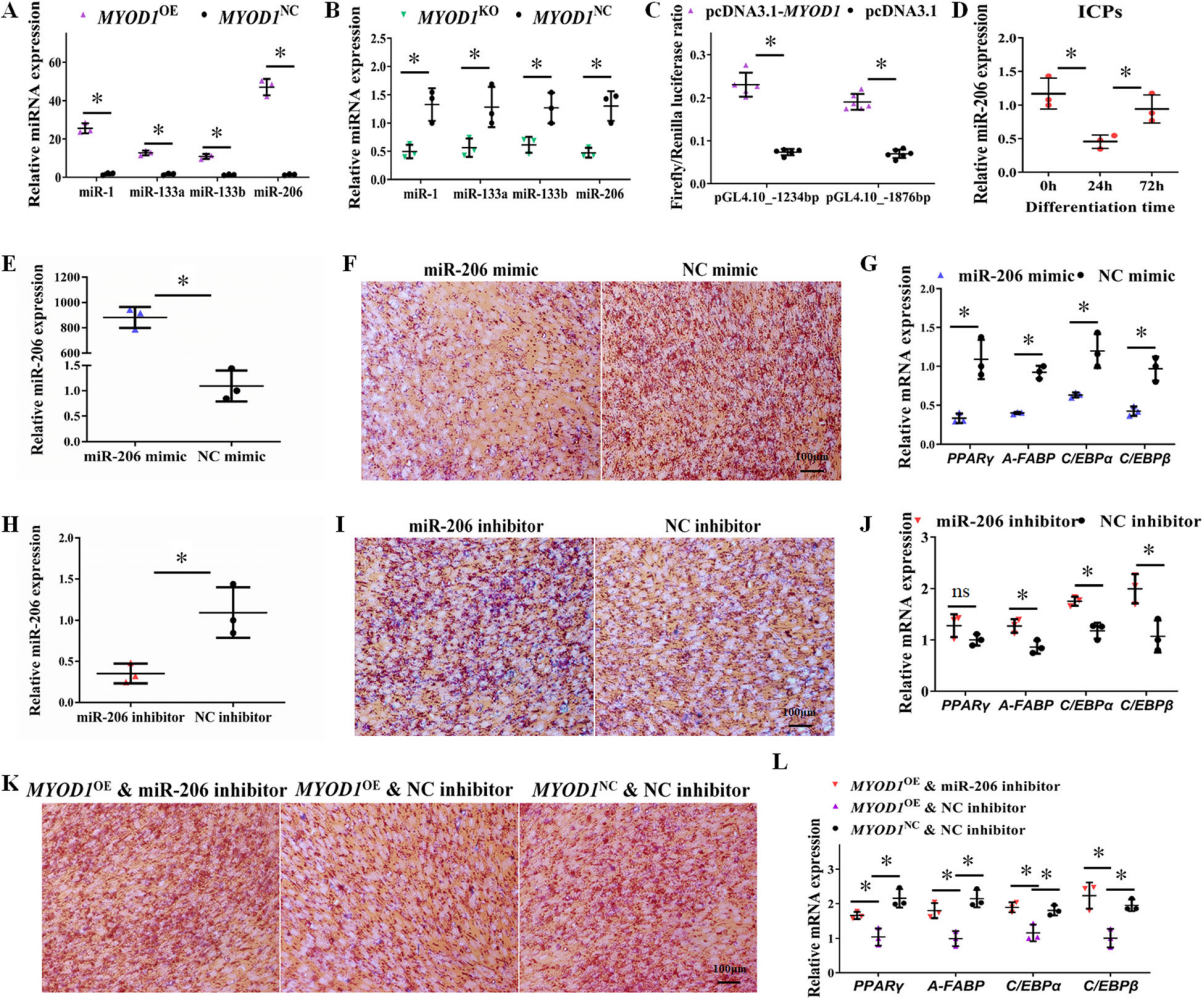
The inhibitory effect of *MYOD1* on adipocyte differentiation was achieved by its downstream gene miR-206. **a** Over-expression of *MYOD1* up-regulated miR-1, miR-133a, miR-133b, and miR-206 expression in ICPs. **b** *MYOD1* knockout down-regulated miR-1, miR-133a, miR-133b, and miR-206 expression in ICPs. **c** *MYOD1* over-expression promoted the relative luciferase activity of the pGL4.10_-1234 bp and pGL4.10_-1876 bp reporter in ICPs. **d** Relative miR-206 expression during ICPs differentiation. **e** Transfected with miR-206 mimic significantly promoted miR-206 expression in ICPs. **f** Over-expression of miR-206 reduced the lipid droplet formation by oil red O staining on day 3. **g** mRNA levels of adipocyte marker genes were analyzed with qPCR. **h** Transfected with miR-206 inhibitor significantly reduced miR-206 expression in ICPs. **i** Inhibition of miR-206 promoted the lipid droplet formation by oil red O staining on day 3. **j** mRNA levels of adipocyte marker genes were analyzed with qPCR. **k** Inhibition of miR-206 could counteract the inhibition effect of *MYOD1* over-expression on adipocyte differentiation. **l** mRNA levels of adipocyte marker genes were analyzed with qPCR. Data are shown as mean ± SD of three biological replicates. The *t*-test was used to analyze the statistical differences between groups. *, *P* < 0.05

*MYOD1* has been confirmed to induce highly conserved myomiRs expression by binding to miRNA upstream regions, including miR-1, miR-133a, miR-133b, and miR-206, and these myomiRs are widely directly involved in the inhibition of other pathways [43–47]. qPCR assay found that *MYOD1*^OE^ cells expressed significantly higher levels of miR-1, miR-133a, miR-133b, and miR-206 (Fig. 3a), and miR-206 has the highest expression fold change (about 40 fold). *MYOD1*^KO^ cells also significantly reduce miR-206 expression (Fig. 3b). Also, we found multiple binding sites of *MYOD1* in the 2000 bp upstream region of gga-miR-206, especially within 1200 bp (Table S7). Thus, we hypothesize that miR-206 may be an essential mediator for *MYOD1* to inhibit adipocyte differentiation. To validate the regulatory relationship between *MYOD1* and miR-206 transcription, two distinct lengths of upstream regions (1876 and 1234 bp) of the gga-miR-206 transcription start site were amplified and cloned into pGL4.10 vector to detect the promoter activity. After cotransfecting with pGL-TK and pcDNA3.1-*MYOD1* into ICPs, both of pGL4.10_ −1876 bp and pGL4.10_ −1234 bp showed a significantly increasing promoter activity compared to cotransfected with pGL-TK and pcDNA3.1 (*P* < 0.05) (Fig. 3c). Our results showed that the chicken *MYOD1* can bind to 1200 bp upstream region of gga-miR-206 and promote the transcription activity of miR-206 in ICPs, as previously reported [24].

### miR-206 is a downstream gene of *MYOD1* that inhibits adipocyte differentiation

We then examined the expression and function of miR-206 during adipocyte differentiation. The expression of miR-206, similar to the protein level of *MYOD1*, was significantly down-regulated after differentiation (Fig. 3d), suggesting a synergistic relationship between them, and both of them were involved in this process. Therefore, we transfected miR-206 mimic and inhibitor into ICPs, respectively. Transfection-mediated gene transfer resulted in up to 840-fold elevation in the expression of miR-206 (Fig. 3e). Over-expression of miR-206 also significantly inhibits lipid droplet accumulation and reduced adipocyte marker genes expression (Fig. 3f, g; Fig. S3a, b), whereas the inhibition of miR-206 promotes adipocyte differentiation (Fig. 3h-j; Fig. S4a, b). Together, these results demonstrated that miR-206 could inhibit adipocyte differentiation.

To examine whether *MYOD1* targets miR-206 to regulate adipocyte differentiation, we transfected *MYOD1*^OE^ cells with miR-206 inhibitor and knock-down of miR-206 could counteract the inhibition effect of *MYOD1* over-expression on adipocyte differentiation (Fig. 3k, l; Fig. S5a, b). Similarly, over-expression of miR-206 also significantly inhibited lipid accumulation in *MYOD1*^KO^ cells (Fig. S6a-e). Together, these results suggest that the inhibitory effect of *MYOD1* on adipocyte differentiation was achieved by its downstream gene miR-206.

### *KLF4* is a miR-206 target gene, functioning as an activator of adipocyte differentiation

**Fig. 4:**
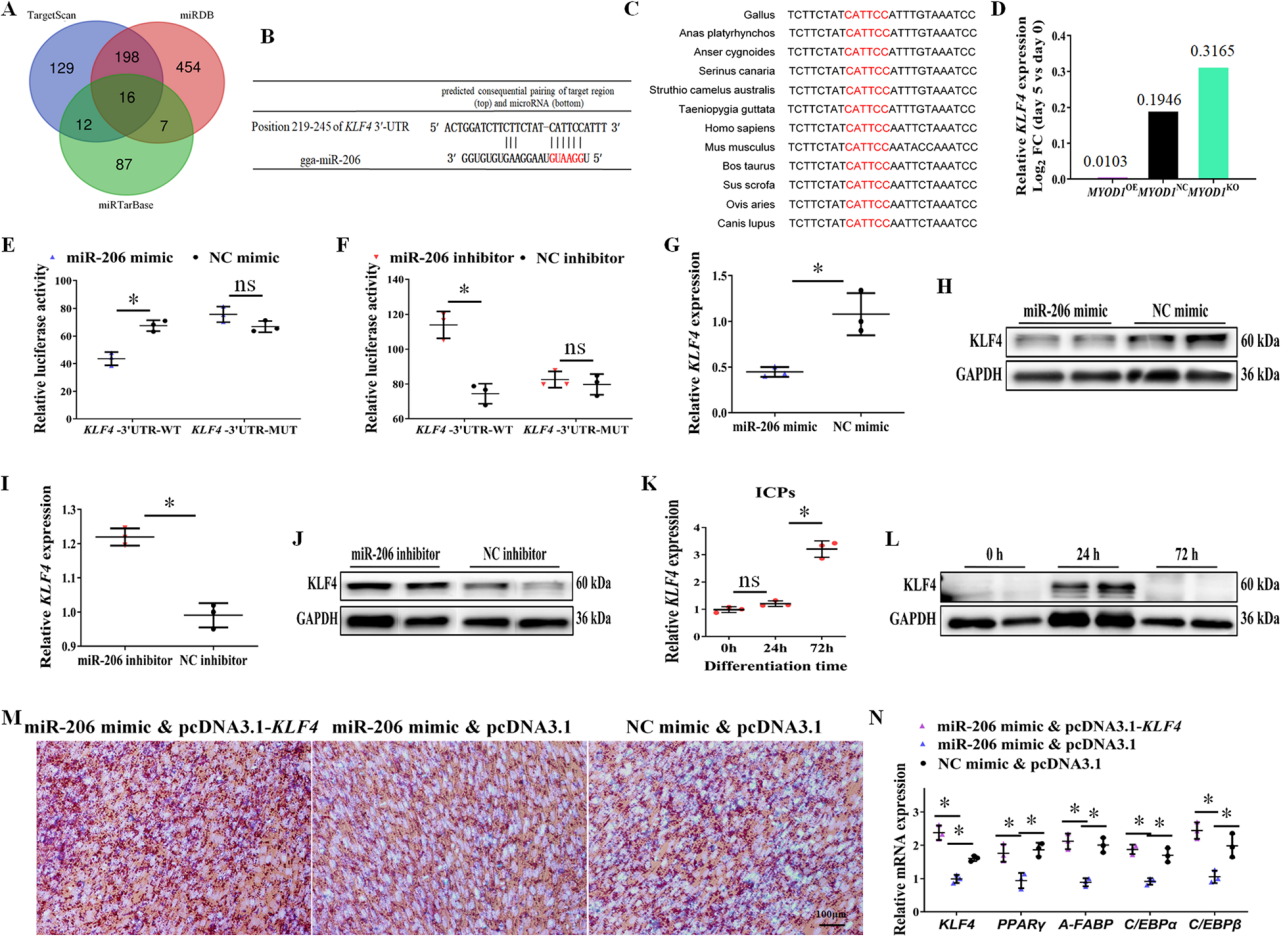
*KLF4* is the miR-206 target gene, functioning as an activator of adipocyte differentiation. **a** Overlap of three miRNA target bioinformatic prediction algorithms. **b** The potential binding site of miR-206 in the *KLF4* mRNA 3′-UTR. **c** The potential binding site (red) of miR-206 in the *KLF4* mRNA 3′-UTR is highly conserved among vertebrates. **d** Compare the fold change of *KLF4* in *MYOD1*^OE^, *MYOD1*^NC^, and *MYOD1*^KO^ cells at day 5 compared to day 0. **e, f** Dual-luciferase reporter assay indicated that miR-206 could bind to the predicted binding site of the *KLF4* mRNA 3′-UTR. **g, h** miR-206 over-expression inhibited *KLF4* mRNA and protein expression in ICPs. **i, j** miR-206 inhibition promoted *KLF4* mRNA and protein expression in ICPs. **k, l** *KLF4* mRNA and protein levels at different time points during ICPs differentiation. **m** Over-expression of *KLF4* could counteract the inhibition effect of miR-206 over-expression on adipocyte differentiation. **n** mRNA levels of adipocyte marker genes were analyzed with qPCR. Data are shown as mean ± SD of three biological replicates. The *t*-test was used to analyze the statistical differences between groups. *, *P* < 0.05

In order to explore the potential mechanism of miR-206 in regulating adipocyte differentiation, we performed bioinformatic analysis. Three bioinformatic tools (TargetScan, miRDB, and miRTarBase) were employed to identify the candidate targets of miR-206 (Table S8). All three programs predicted a total of 16 miR-206 target genes, such as *KLF4*, *CCND2*, and *UTRN* (Fig. 4a). Among them, we noticed that *KLF4*, a key activator of adipocyte differentiation, contains the miR-206 binding site in its 3′-UTR (Fig. 4b). This binding site is conserved among avian (Fig. 4c), suggesting the biological relevance of miR-206 in regulating *KLF4* expression in avian. mRNA-seq also showed that the fold change (day 5 vs. day 0) of *KLF4* expression was the highest in the differentiated *MYOD1*^KO^ cells, followed by the *MYOD1*^NC^, and the lowest in the *MYOD1*^OE^ cells (Fig. 4d). To validate whether *KLF4* is the target gene of miR-206, the 3′-UTR of chicken *KLF4* containing the wild-type or mutated miR-206-binding sites were cloned into pmirGLO vector, and the luciferase activity was found to be significantly decreased in ICPs cotransfected with the vector carrying the wild-type-binding site in the presence of miR-206 mimics, but not in ICPs carrying the mutated-binding site (Fig. 4e). On the other hand, the luciferase activity was found to be significantly increased in ICPs cotransfected with the vector carrying the wild-type-binding site in the presence of miR-206 inhibitor, but not in ICPs carrying the mutated-binding site (Fig. 4f). In addition, over-expression of miR-206 inhibited the mRNA and protein level of KLF4 (Fig. 4g, h), and the inhibition of miR-206 promoted the mRNA and protein level of KLF4 (Fig. 4i, j). Therefore, the above results indicated that *KLF4* is the miR-206 target gene.

During adipocyte differentiation, *KLF4* mRNA has gradually up-regulated its expression until day 3 (Fig. 4k). However, the protein level for *KLF4* reached the highest on the day 1 after differentiation and disappeared on day 3 (Fig. 4l). Our resultsalso showed that *KLF4* was induced very early following the induction of adipogenesis, and function as an early regulator of adipocyte development has been attributed to its capacity to induce C/EBPβ expression as the previous report [30]. Furthermore, we cotransfected miR-206 and pcDNA3.1-KLF4 into ICPs, and *KLF4* over-expression can counteract the inhibition effect of miR-206 on adipocyte differentiation (Fig. 4m, n; Fig. S7a, b). Therefore, *KLF4* is the miR-206 target gene, which can function as an activator of adipocyte differentiation.

**Fig. 5:**
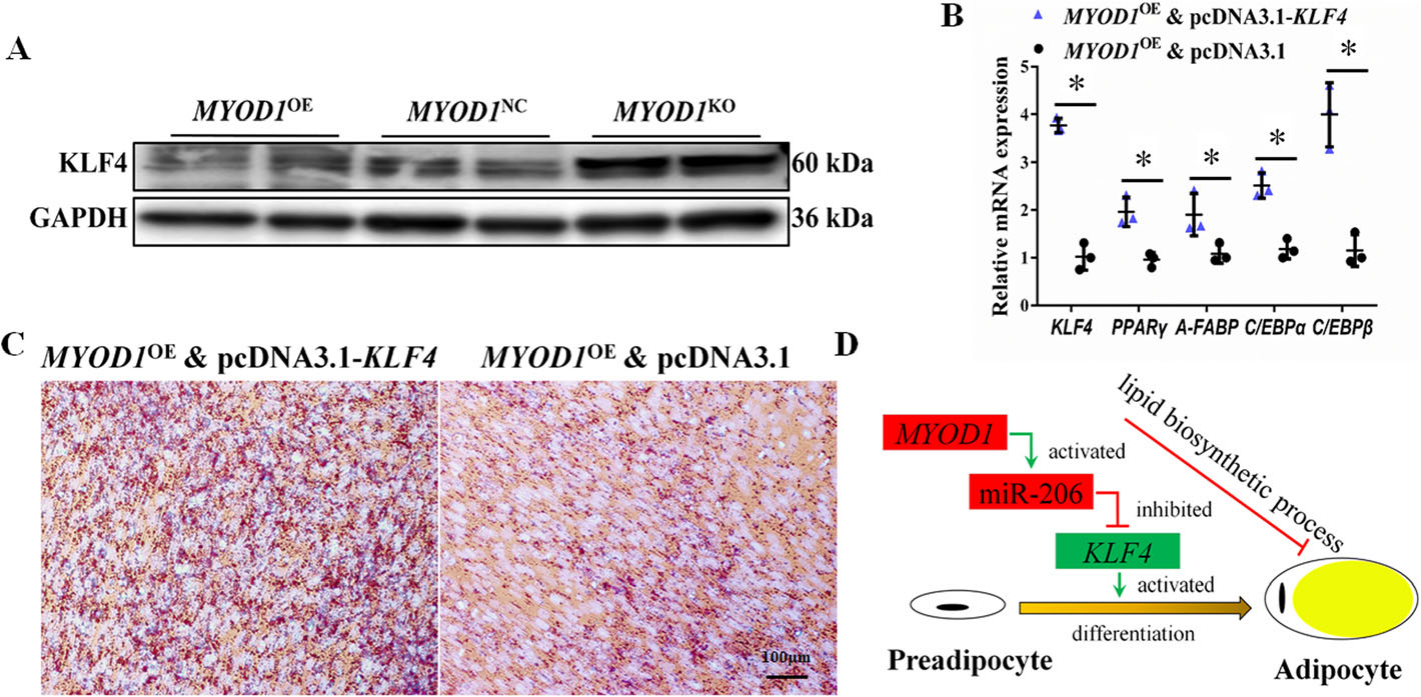
*MYOD1* affected *KLF4* expression. **a** Western blot analysis of the protein levels of *KLF4* in *MYOD1*^OE^, *MYOD1*^NC^, and *MYOD1*^KO^ cells. **b** mRNA levels of adipocyte marker genes were analyzed with qPCR. **c** Representative images of *KLF4* over-expression promoted the lipid droplet formation by oil red O staining (red). **d** Model of the *MYOD1*-mediated regulatory pathway for adipocyte differentiation. Data are shown as mean ± SD of three biological replicates. The t-test was used to analyze the statistical differences between groups. *, *P* < 0.05

Based on the above results, we can deduce *MYOD1* inhibits adipocyte differentiation by inhibiting the expression of *KLF4*. Western blot show *MYOD1*^KO^ cells expressed significantly higher protein levels of *KLF4*, while *MYOD1*^OE^ cells expressed lower protein levels of *KLF4* (Fig. 5a). Finally, over-expression of *KLF4* also significantly promoted adipocyte differentiation in *MYOD1*^OE^ cells (Fig. 5b, c; Fig. S8a, b). Also, over-expression of *MYOD1* could significantly inhibit the expression of *KLF4*’s target genes *C/EBPβ*, while inhibiting miR-206 or over-expression of *KLF4* in *MYOD1*^OE^ cells will significantly increase *C/EBPβ* expression.

## Discussion

Avian are important farm animals throughout the world, producing eggs and high-quality meat for humans. Muscle mass and fat content are both important traits for meat-producing chickens but attaining an appropriate muscle and fat ratio is an excellent challenge for the broiler industry [48]. Discovering functional genes that can simultaneously regulate muscle and adipocyte development is a key step to improve these traits [49]. Previous and current transcriptomic data have shown that many muscle development genes were differentially expressed during adipocyte differentiation. Among these, our study investigated *MYOD1* inhibited avian adipocyte differentiation via the miRNA-206/*KLF4* axis (Fig. 5d). We have also identified a negative regulation between *MYOD1* and lipid biosynthesis genes during adipogenesis. Previous studies reported that *MYOD1* expression in brown fat is significantly reduced, and it inhibits brown fat development through *PRDM16* [50]. A lineage-tracing study reveals that the *MYOD1* lineage does not give rise to brown adipocytes, indicating a role of *MYOD1* in myogenic cell fate switch in the common progenitors that give rise to both myoblasts and brown preadipocytes [20]. Moreover, the loss of *MYOD1* also facilitates the adipogenic trans-differentiation of C2C12 myoblasts by the miR-133/*IGF1R*/PI3K/AKT signaling pathway [47]. These findings revealed a novel function of *MYOD1* in adipogenesis.

*MYOD1* is considered as a master regulator of myogenesis as its expression can induce myogenic differentiation in myoblasts, fibroblasts, and a variety of other cell types [18, 51, 52]. In this study, muscle structure development was significantly enriched in the up-regulated DEGs of *MYOD1*^OE^ cells, and also enriched in down-regulated DEGs of *MYOD1*^KO^ on day 0, which all demonstrate the strong transcriptional regulatory activity of *MYOD1* on downstream genes. Whether the ectopic expression of *MYOD1* in preadipocytes promoted the myogenesis has not been examined in this study. Still, this evidence makes us believe that the over-expression of *MYOD1* can promote the trans-differentiation of preadipocytes into muscle cells. In addition, several co-culture experiments have revealed that adipogenesis is strongly inhibited by the presence of satellite cell-derived myofibres [8, 53]. Supportively, the transcriptomic analysis also suggested that *MYOD1* would be a repressor of adipogenesis by inhibiting the expression of lipid biosynthesis genes. Similarly, myogenesis was wholly blocked in both the *MYOD1*/*MYF5* and *MYOD1*/*IGF2* double knockout mice. Both results showed potential functions of *MYOD1* on adipogenesis [54, 55]. On the other hand, myoblasts trans-differentiate into mature adipocytes by ectopic expression of adipogenic transcription factors under conditions permissive for adipogenesis [14]. The mutual exclusion of the two lineage-specific transcription factors balances and determines the developmental separation of fat and muscle tissue.

The current fast-growing and high-energy diet makes the chicken more likely to show myopathy, such as white striping, characterized by more fat in the breast muscles, resulting in meat with higher fat content and lower protein content [56, 57]. Combined with previously reported that *MYOD1* can promote muscle development, increasing the expression of *MYOD1* may be an effective strategy to treat these diseases.

Finally, we show that miR-206 is an important mediator of *MYOD1* induced inhibition of adipogenesis. Previous study has proven that miR-206 inhibits adipocyte differentiation by targeting *c-MET* and its downstream PI3K/AKT pathway in mammals [28, 29]. Through TargetScan and miRDB analysis, however, *c-MET* was not the target gene of gga-miR-206 (Table S8), and the potential binding site of gga-miR-206 is also not in the 3′-UTR of *c-MET* (Table S9), which may be due to a divergence in evolution. Studies have shown that *KLF4* expression is required for *C/EBPβ* expression observed in the early stages of adipogenesis. Indeed, in this study, we found that *C/EBPβ* and its downstream genes *C/EBPα* and *PPARγ* were significantly inhibited in *MYOD1*^OE^ cells than in *MYOD1*^NC^, but they were increased after over-expression of *KLF4* or inhibition of miR-206, suggesting a possible involvement of *KLF4* and *C/EBPβ* in the inhibition of *MYOD1* on *C/EBPα* and *PPARγ* transcription. However, given the over-expression of *MYOD1* also promotes other myomiRs (miR-1, miR-133a, miR-133b) expression, we are implying that miRNA-206 may not be the sole target of *MYOD1* to inhibit adipocyte differentiation. The inhibitory effect of *MYOD1* on adipocyte differentiation should probably be accounted for by multiple targets. Therefore, identifying *MYOD1*’s other targets and understanding how it may affect these targets will warrant further investigation.

## Conclusions

In summary, this study provides new insight into that *MYOD1* also works as the repressor of adipocyte differentiation via miR-206/*KLF4* axis in avian adipocyte model. Considering the significant role of adipocyte differentiation in the formation and function of adipose, clarification of the mechanism of *MYOD1*-mediated regulation of adipocyte differentiation is essential for exploring strategies for the treatment of metabolic disorders, including white striping. In combination with previous findings of the beneficial role of *MYOD1* in muscle differentiation, we proposed that *MYOD1* may be a crucial target for improving the ratio of muscle to fat in avian.

## Supplementary Information

**Additional file 1:** **Table S1.** Primer sequences used for the PCR and qPCR analysis.

**Additional file 2:** **Fig. S1.** Statistics of the number and size of lipid droplets per cell.

**Additional file 3:** **Table S2.** List of all DEGs in MYOD1^OE^ cells vs. MYOD1^NC^ cells and MYOD1^KO^ cells vs. MYOD1^NC^ on day 0.

**Additional file 4:** **Table S3.** GO analysis of DEGs in MYOD1^OE^ cells vs. MYOD1^NC^ cells and MYOD1^KO^ cells vs. MYOD1^NC^ on day 0.

**Additional file 5:** **Table S4.** List of all DEGs before (day 0) and after adipogenic differentiation (day 5) in MYOD1^KO^, MYOD1^NC^ and MYOD1^OE^ cells.

**Additional file 6:** **Table S5.** GO analysis of DEGs before (day 0) and after adipogenic differentiation (day 5) in MYOD1^KO^, MYOD1^NC^ and MYOD1^OE^ cells.

**Additional file 7: Table S6.** A list of the expression fold changes of known lipid biosynthesis genes in MYOD1^KO^, MYOD1^NC^ and MYOD1^OE^ cells. (Genes are selected from genecards database: Pathcards: Fatty Acyl-CoA Biosynthesis & Triglyceride Biosynthesis).

**Additional file 8:** **Table S7.** Binding sites of MYOD1 in the 2000 bp upstream region of gga-miR-206.

**Additional file 9:** **Table S8.** Predicted target genes of gga-miR-206.

**Additional file 10:** **Table S9.** Predicted binding miRNAs on 3′-UTR of c-Met gene.

## Data Availability

The data analyzed during the current study are available from the corresponding author on reasonable request.

## Abbreviations

ADORA2B: A2b adenosine receptor
A-FABP: Adipocyte-type fatty acid-binding protein
CCND2: Cyclin D2
CDS: Coding sequence
C/EBPs: CCAAT enhancer binding proteins
c-MET: MET proto-oncogene, receptor tyrosine kinase
CRISPR: Clustered regularly interspaced short palindromic repeats
DEGs: Differentially expressed unigenes
FBS: Foetal bovine serum
GO: Gene Ontology
IGF1R: Insulin-like growth factor 1 receptor
IGF2: Insulin-like growth gactor 2
KLF4: Kruppel-like factor 4
MEF2C: Myocyte enhancer factor 2C
MYF5: Myogenic factor 5
MYOD1: Myogenic differentiation 1
MYOG: Myogenin
PPARγ: Peroxisome proliferator activated receptor gamma
PRDM16: PR/SET domain 16
RNA-seq: High-throughput sequencing of RNA
RT-qPCR: Quantitative real-time PCR
UTR: Untranslated region
UTRN: Utrophin
