# Supplementary material for: MYOD1 inhibits avian adipocyte differentiation via miRNA-206/KLF4 axis
Source: J Anim Sci Biotechnol. 2021 May 5;12:55. doi: 10.1186/s40104-021-00579-x (PMC8101123; doi:10.1186/s40104-021-00579-x)

**Supplementary Information**

**Fig. S1：MYOD1^OE^ cells reduced the number and size of lipid droplets (LDs) compare to MYOD1^NC^ cells. (a)** The number of LDs. **(b)** The average size of LDs. Data are shown as mean ± SD. The independent sample t-test was used to analysis the statistical differences between groups; n=4 for **(a)** and n=100 for **(b)**; *p<0.05.

**
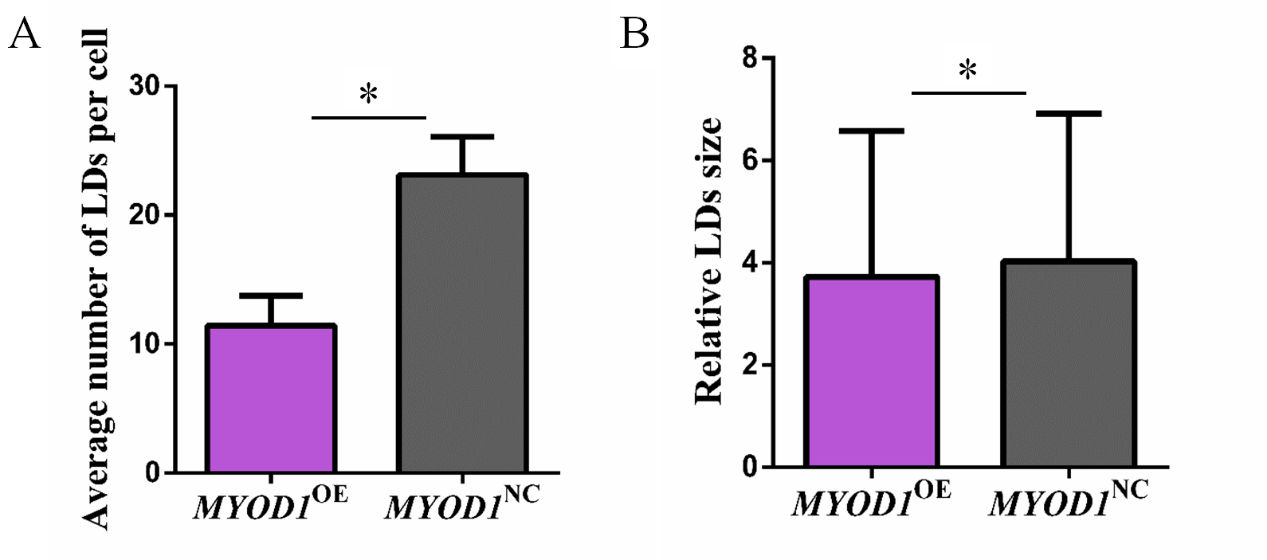
**

**Fig. S2：MYOD1^KO^ cells increases the number and size of LDs compare to MYOD1^NC^ cells. (a)** The number of LDs. **(b)** The average size of LDs. Data are shown as mean ± SD. The independent sample t-test was used to analysis the statistical differences between groups; n=4 for **(a)** and n=100 for **(b)**; *p<0.05.


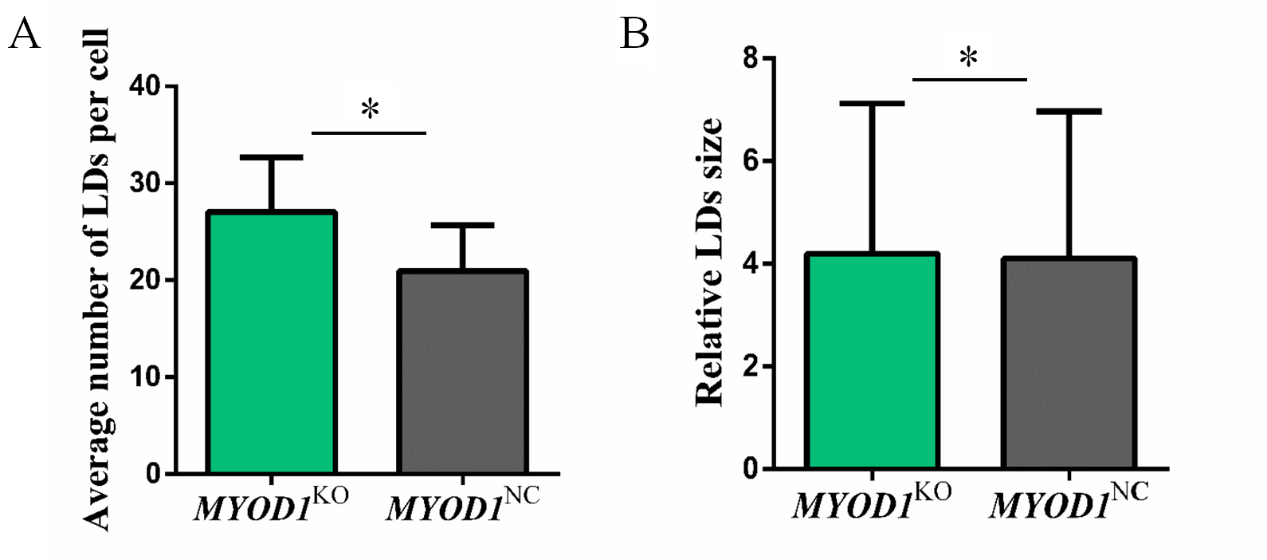


**Fig. S3：Over-expression of miR-206 reduced the lipid droplet formation. (a)** The number of LDs. **(b)** The average size of LDs. Data are shown as mean ± SD. The independent sample t-test was used to analysis the statistical differences between groups; n=4 for **(a)** and n=100 for **(b)**; *p<0.05.

**
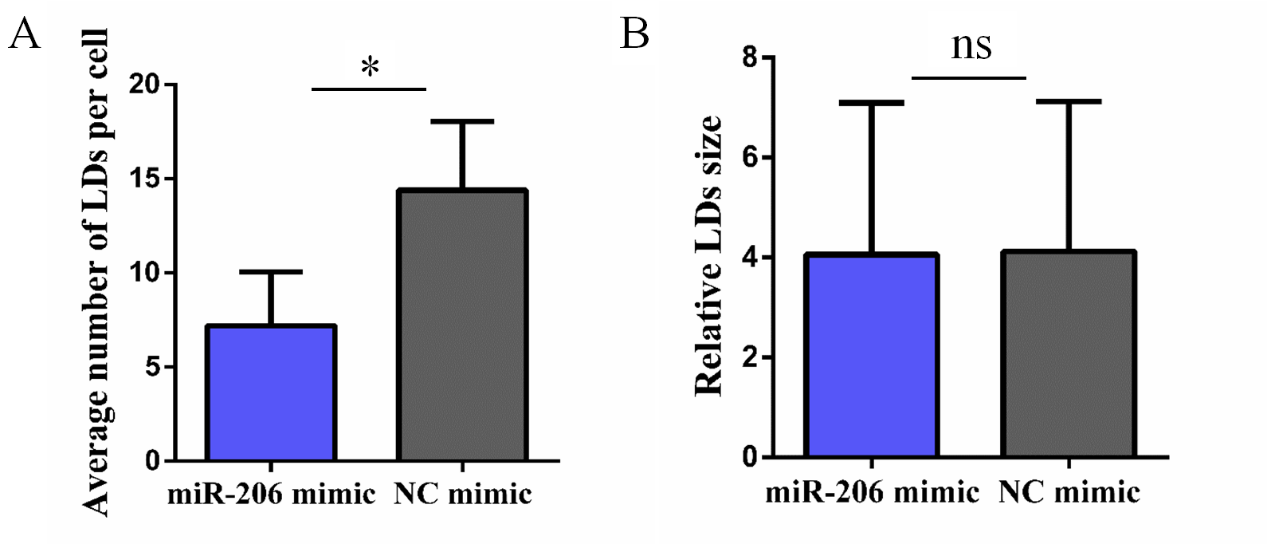
**

**Fig. S4：Inhibition of miR-206 promoted the lipid droplet formation. (a)** The number of LDs. **(b)** The average size of LDs. Data are shown as mean ± SD. The independent sample t-test was used to analysis the statistical differences between groups; n=4 for **(a)** and n=100 for **(b)**; *p<0.05.


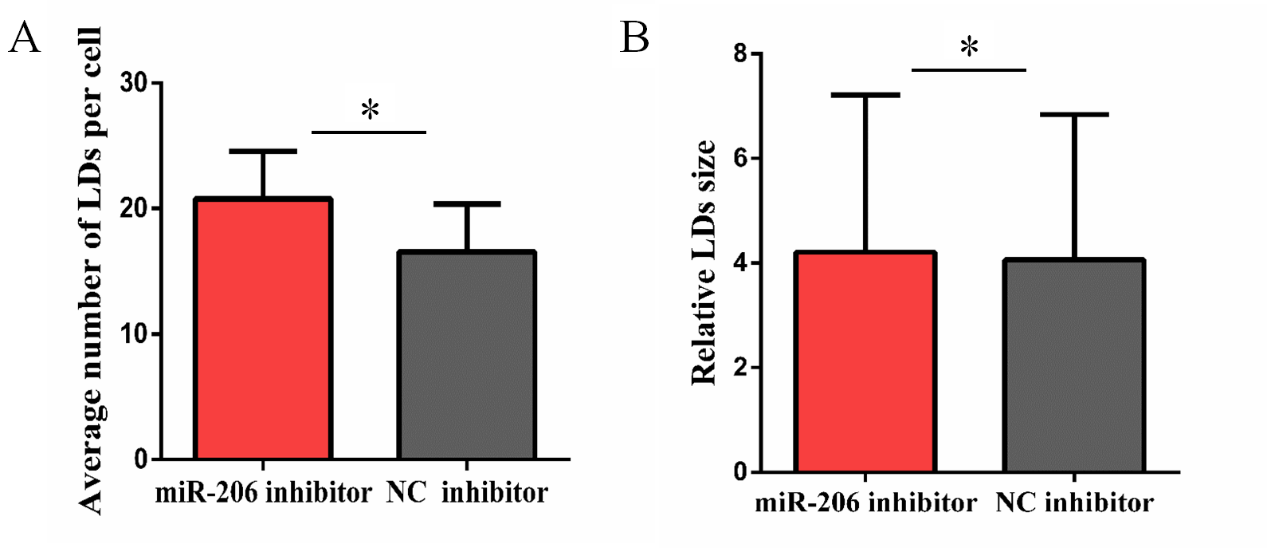


**Fig. S5：Inhibition of miR-206 could counteract the inhibition effect of MYOD1 over-expression on the lipid droplet formation. (a)** The number of LDs. **(b)** The average size of LDs. Data are shown as mean ± SD. The independent sample t-test was used to analysis the statistical differences between groups; n=4 for **(a)** and n=100 for **(b)**; *p<0.05.


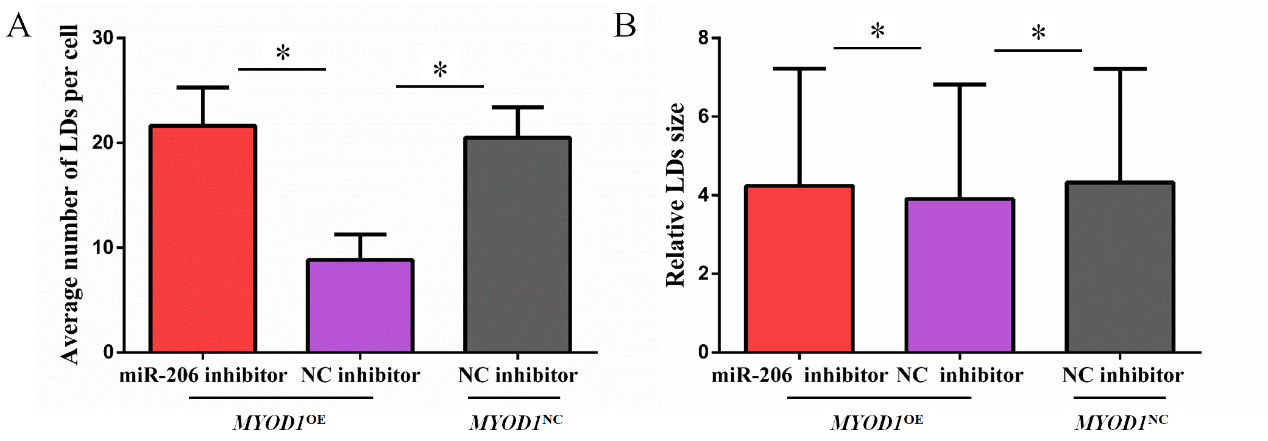


**Fig. S6：Over-expression of miR-206 significantly inhibited lipid droplet formation in MYOD1^KO^ cells. (a)** Transfected with miR-206 mimic significantly increased miR-206 expression in MYOD1^KO^ cells. **(b)** mRNA levels of adipocyte marker genes were analyzed with qPCR (n=3). **(c)** Representative images of miR-206 over-expression inhibited the lipid droplet formation in MYOD1^KO^ cells by Oil Red O staining. **(a)** The number of LDs (n=4). **(b)** The average size of LDs (n=100). Data are shown as mean ± SD of three biological replicates. The independent sample t-test was used to analysis the statistical differences between groups. *, *P* ＜ 0.05.


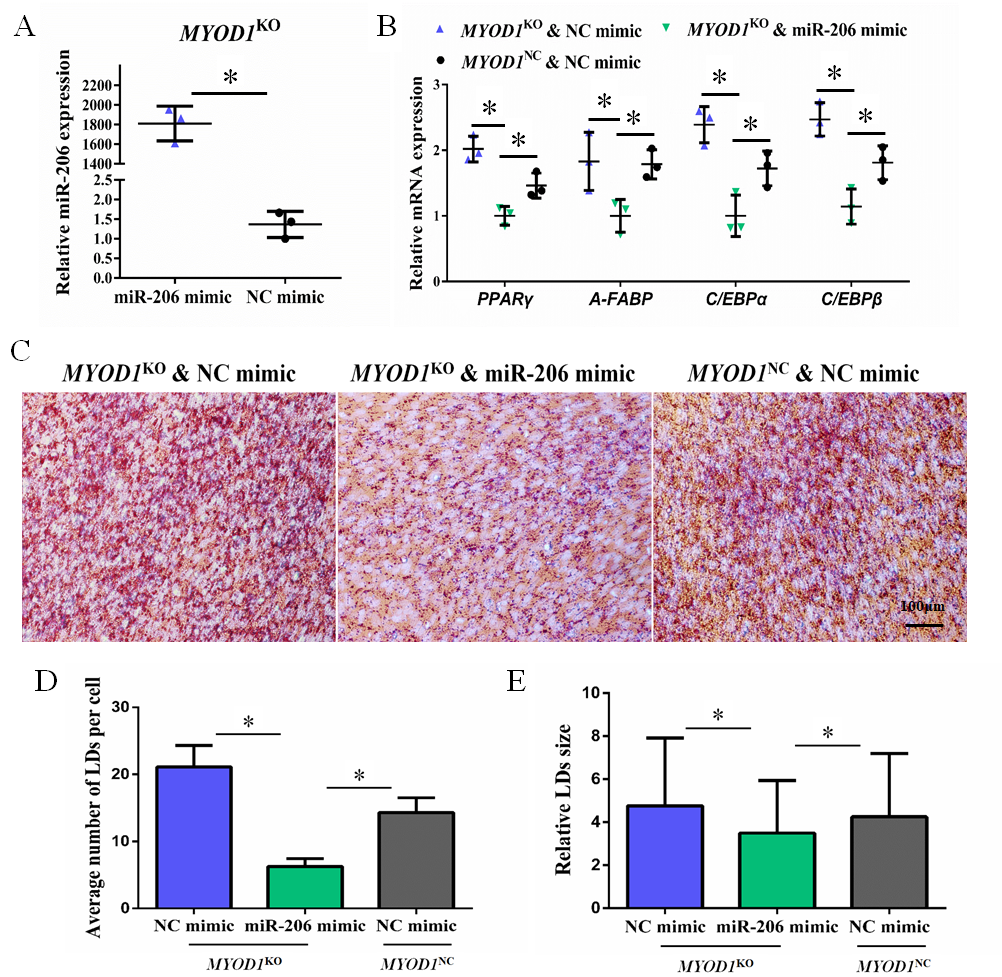


**Fig. S7：Over-expression of *KLF4* could counteract the inhibition effect of miR-206 over-expression on the lipid droplet formation. (a)** The number of LDs. **(b)** The average size of LDs. Data are shown as mean ± SD. The independent sample t-test was used to analysis the statistical differences between groups; n=4 for **(a)** and n=100 for **(b)**; *p<0.05.


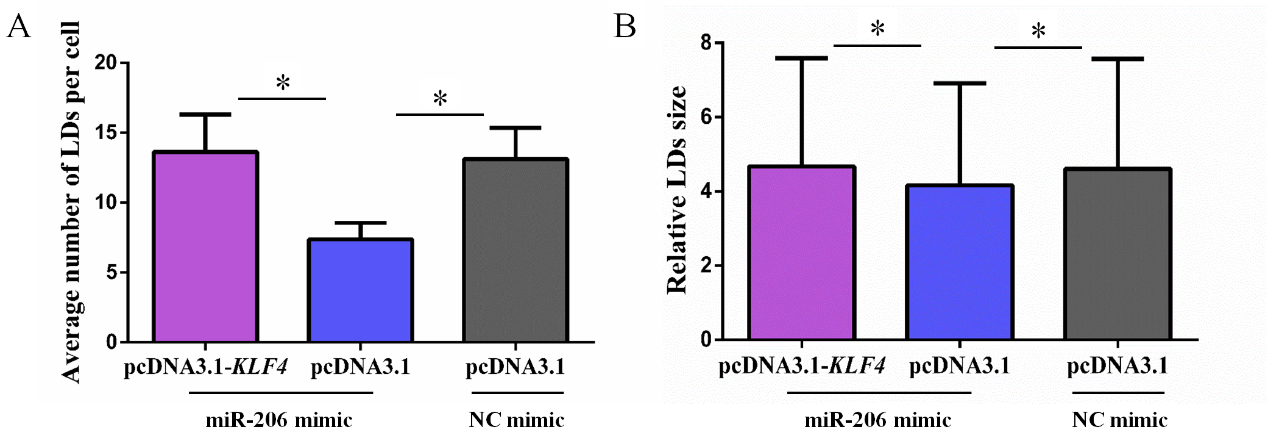


**Fig. S8：Over-expression of *KLF4* could counteract the inhibition effect of *MYOD1* over-expression on the lipid droplet formation. (a)** The number of LDs. **(b)** The average size of LDs. Data are shown as mean ± SD. The independent sample t-test was used to analysis the statistical differences between groups; n=4 for **(a)** and n=100 for **(b)**; *p<0.05.


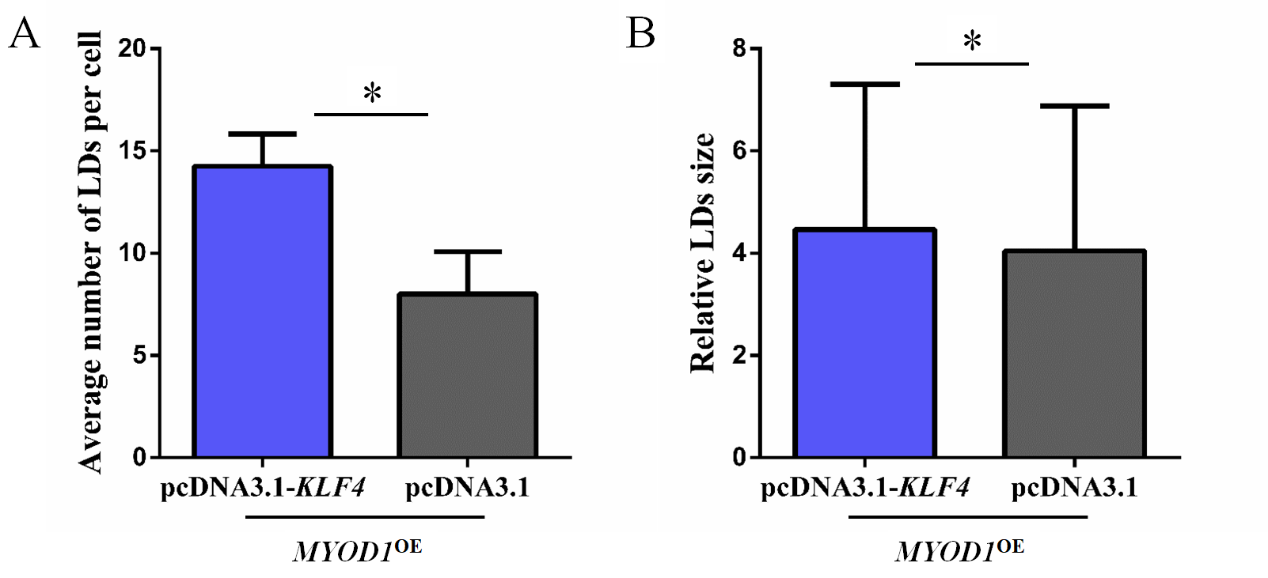

Supplement: Supplementary file 2 — Additional file 2: Fig. S1. Statistics of the number and size of lipid droplets per cell. [file 40104_2021_579_MOESM2_ESM.docx]
